# Comparison of conventional IVF versus ICSI in non-male factor, normoresponder patients

**Published:** 2012-03

**Authors:** Maryam Eftekhar, Farnaz Mohammadian, Fariba Yousefnejad, Behnaz Molaei, Abbas Aflatoonian

**Affiliations:** 1 *Department of Obstetrics and Gynecology, Research and Clinical Center for Infertility, Shahid Sadoughi University of Medical Sciences, Yazd, Iran.*; 2 *Department of Obstetrics and Gynecology, Zanjan University of Medical Sciences, Azadi Avenue, Zanjan, Iran. *

**Keywords:** *Infertility*, *ICSI*, *In-vitro fertilization*, *Fertilization*, *Pregnancy rate*

## Abstract

**Background: **Conventional IVF and ICSI are two common techniques to achieve fertilization. IVF has long been used for treatment of infertility, although it is not an effective treatment in severe male infertility. The use of ICSI has been expanded in severe male factor and fertilization failure after IVF cycle. In spite of the widespread use of ICSI in patients with non-male factor infertility, there is still little evidence to confirm its effectiveness in this population.

**Objective: **To evaluate assisted reproductive technology outcomes between IVF and ICSI cycles in non-male factor, normoresponder patients.

**Materials and Methods: **A total of 220 non-male factors, normoresponder patients who were indicated for ART were enrolled in this study. The patients received standard long GnRH agonist or GnRH antagonist protocols for ovarian stimulation and after oocytes retrieval, the patients were divided into two groups (IVF and ICSI groups). In IVF group (n=112), all of retrieved oocytes were treated by conventional IVF and in ICSI group (n=88), microinjection (ICSI) was done on all of retrieved oocytes.

**Results:** In IVF group, fertilization and implantation rates were significantly higher than ICSI group (66.22% and 16.67% in IVF group versus 57.46% and 11.17% in ICSI group, respectively). Chemical and clinical pregnancy rates were statistically higher in IVF group as compared with the ICSI group (42.9% vs. 27.3% and 35.7% vs. 21.5%, respectively).

**Conclusion:** According to our study, the routine use of ICSI is not improved fertilization, implantation and chemical pregnancy rates and is not recommended in non-male factor, normozoospermic patients.

## Introduction

Conventional IVF and ICSI are two common techniques to achieve fertilization. In-vitro fertilization (IVF) has long been used for treatment of infertility. Although it has made an important role in the treatment of female infertility, it is not an effective treatment in severe male infertility.

Intracytoplasmic sperm injection (ICSI) is an assisted fertilization procedure that has been introduced in 1992. Conventional IVF contains standard insemination and ICSI involves injection of single spermatozoa into a mature oocyte (1-7). 

Conventional IVF was much less effective when the semen characteristics were grossly below the standard values regarding to concentration, morphology or motility and when fertilization rates in previous cycles have been low (1, 8-12). The risk of complete fertilization failure after conventional IVF was estimated 12.5% in normozoospermia and tubal factor infertility, 16.7% in unexplained infertility and up to 50% in astenozoospermia (5, 13-14). 

Gamete micromanipulation is the suitable method to overcome this problem in these cases. Recently, the use of ICSI has been expanded in fertilization failure after ejaculatory dysfunction and immunological infertility (2, 4, 8, 11, 15). The safety of ICSI is still unknown and the unnecessary ICSI appears to make higher cost, increased time and unethical method, however ICSI is very successful in treatment of severe male infertility. In spite of, the widespread use of ICSI in patients with non-male factor infertility, there is a little evidence to confirm its effectiveness in this population (11, 16-17). 

The aim of this study was to compare assisted reproductive technology (ART) outcomes between IVF and ICSI cycles in non-male factor, normoresponder patients.

## Materials and methods

This study was a cross-sectional study including patients who were scheduled for ART from April 2009 to September 2010. The study was approved by ethics committee of Research and Clinical Center for Infertility, Shahid Sadoughi University of Medical Sciences.

Women with basal FSH ≥ 10 IU/ml, age > 38 years, previous IVF failure ≥ 3 and history of pelvic surgery were excluded from the study. Normozoospermic couple with a sperm count >10 million/ml, normal morphology ≥ 8% (Kruger’s strict criteria), and progressive motile sperm ≥40% participated in the study.

All of the patients that were included in the study were divided into group I (IVF group, n=122) and group II (ICSI group, n=110). Controlled ovarian stimulation was done using down-regulation with gonadotropin-releasing hormone (GnRH) agonist protocol with urinary or recombinant FSH or GnRH antagonist protocol with urinary or recombinant FSH. 

When at least two follicles reached a mean diameter of 18 mm, using transvaginal ultrasonography, 10000 IU HCG was administrated and oocytes retrieval was carried out 36 hours after HCG injection. The patients were excluded from the study when retrieval oocytes were lower than 5 or more than 15.

In group I, total retrieved oocytes were treated by conventional IVF and were inseminated 4 hours after oocytes retrieval with 60000 motile sperm in 1 ml of IVF medium. In group II, total retrieved oocytes were treated by ICSI. Immediately before oocyte micromanipulation, cumulus and corona cells were removed enzymatically by incubating the oocytes in 1 ml of IVF medium containing 80 IU/ml hyaluronidaze for 2-3 min. After selection of mature oocytes (metaphase II), a single motile spermatozoa with apparently normal morphology was microinjected into the ooplasm at the 3 o’clock position. 

Fertilization was evaluated 16-18 hours after IVF or ICSI. Normal fertilization was defined as zygotes with two pronuclei (2PN). Zygotes with 2PN were cultured and embryos were transferred using a Labotect catheter (Labotect, Gottingen Germany) 48-72 hours after oocytes retrieval. Luteal phase support was started with progesterone in oil (Progesterone, Aburaihan Co., Tehran, Iran) 100 mg daily IM on the day of oocyte retrieval and was continued until the documentation of fetal heart activity by ultrasound. 

Chemical pregnancy was defined by positive beta-hCG 14 days after embryos transfer. Clinical pregnancy was identified as observation of fetal heart activity by transvaginal ultrasonography that was performed 3 weeks after positive beta-hCG.

**Statistical analysis**

Statistical analysis was performed using the statistical package for the social science version 15.0 for windows (SPSS Inc., Chicago, IL, USA). Differences among variables were analyzed using the Student’s t-test, Mann-Whitney, and chi-squared tests. P-value of less than 0.05 was considered statistically significant.

## Results

232 couples were participated in this study and the patients were divided into two groups. 122 couples were enrolled in IVF group and 110 couples were enrolled in ICSI group. 12 patients did not start treatment (4 patients in IVF group and 8 patients in ICSI group). 220 patients started ovarian stimulation (118 patients in IVF group and 102 in ICSI group). 

In IVF group 6 patients and in ICSI group 14 patients were excluded from the study because of retrieved oocytes were less than 5 or more than 15. Finally IVF was done on 112 cycles and ICSI was done on 88 cycles. 

Basic characteristics of patients are summarized in Table I. Etiology of infertility was comparable between two groups (Table II). There was no statistically significant difference regarding to total used gonadotropins ampoules, duration of stimulation, number of retrieved oocytes and number of obtained embryos in the two groups (Table III). 

In IVF group, fertilization and implantation rates were significantly higher than ICSI group (66.22% and 16.7% in IVF group versus 57.46% and 11.17% in ICSI group, respectively). As can be seen in table 4, chemical and clinical pregnancy rates were statistically higher in IVF group as compared with the ICSI group (42.9% vs. 27.3% and 35.7% vs. 21.5%, respectively). 

**Table I:** Basic characteristics of patients of patients in two groups

**Variables**	**IVF ** a **group**	**ICSI** b **group**	**p-value**
Female age (years)	29.14 ± 4.2	29.45 ± 3.4	0.576
Duration of infertility (years)	8.64 ± 5.3	7.95 ± 3.7	0.305
Basal FSH (IU/L)	6.40 ± 3.8	7.23 ± 2.8	0.164

a : In Vitro Fertilization.

b : Intra Cytoplasmic Injection.

**Table II:** Etiology of infertility in two groups

**Variable**	**IVF group**	**ICSI group**	**p-value**
Ovarian, n (%)	31 (27.6%)	26 (29.5%)	0.321
Tubal, n (%)	17 (15.1%)	15(17%)	0.745
Mild endometriosis, n (%)	13 (11.6%)	5 (5.6%)	0.084
Unexplained, n (%)	36 (32.1%)	30 (34%)	0.091
Uterine, n (%)	1 (0.89%)	1 (1.1%)	0.380
Mixed, n (%)	14 (12.5%)	11 (12.5%)	0.127
Total, n (%)	112 (100%)	88 (100%)	

**Table III:** Results of ovarian stimulation in two groups

**Variables**	**IVF group**	**ICSI group**	**p-value**
No. of used gonadotropins ampoules	29.70 ± 7.2	29.50 ± 8.9	0.818
Duration of stimulation (days)	11.14 ± 1.6	11.40 ± 2.1	0.313
No. of retrieved oocytes	7.21 ± 1.2	7.13 ± 1.6	0.703
No. of obtained embryos	4.28 ± 2.6	3.72 ± 2.4	0.129
No. of transferred embryos	2.23 ± 5.8	2.38 ± 0.5	0.185

**Table IV:** ART outcome in two groups

**Variables**	**IVF group**	**ICSI group**	**p-value**
Fertilization rate (%)	66.22%	57.46%	0.036
Implantation rate (%)	16.67%	11.17%	0.049
Chemical pregnancy rate, n (%)	48 (42.9%)	24 (27.3%)	0.026
Clinical pregnancy rate, n (%)	40 (35.7%)	19 (21.5%)	0.031

## Discussion

Although the use of ICSI has been estimated as highly advanced procedure in the treatment of male infertility, recently there is a trend to use of this technique for non-male factor infertility (17). However the mechanical damage to the oocytes after ICSI may cause a detrimental effect and decline the chances of fertilization and pregnancy (7). 

According to our results, fertilization and implantation rates were significantly higher In IVF group than ICSI group (66.22% and 16.67% in IVF group vs. 57.46% and 11.17% in ICSI group, respectively). Chemical and clinical pregnancy rates were statistically higher in IVF group as compared with the ICSI group (42.9% vs. 27.3% and 35.7% vs. 21.5%, respectively). We found the superiority of IVF compared to ICSI and also we found that the use of ICSI did not improved fertilization, implantation and clinical pregnancy rates in non-male factor, normoresponder patients. 

Results of Bhatlachara’s study failed to support benefit of ICSI over IVF in non-male factor subfertility (8). Contrary to their study, we randomized IVF or ICSI procedures based on cycles instead of sibling oocytes, thus assessment of clinical outcomes such as implantation and pregnancy rates were possible in our study.

Similar to our study, Howward *et al* concluded that using ICSI in non-male factor patients was not associated with improved fertilization, pregnancy, or live birth rates and also Bhattacharya *et al* study did not show better ICSI outcome in non-male factor infertility and their results supported the use of ICSI only for severe male factor problems (8, 17).

When there is a history of fertilization failure in normozoospermic patients, performing of ICSI may lead to higher fertilization and pregnancy rates (18-19). Plachot *et al* concluded that ICSI procedure in sibling oocytes prevents the cancellation of embryo transfer following complete fertilization failure with conventional IVF (16). Ou *et al* reported higher fertilization rate with ICSI in low oocytes retrieval cycles and suggested this technique may be better than conventional IVF in these cases (6). 

However our research was done in normoresponder patients and cycles with retrieved oocytes lower than 5 were excluded from the study. Some studies showed lower blastocysts formation in ICSI versus IVF procedures. 

Biological differences between the process of fertilization during ICSI and conventional IVF may contribute to decrease of blastocyst formation in ICSI cycles; The reasons for this comment may be the harmful effects of ICSI on oocyte and the further development of embryo or the inaccurate positioning of injection needle in regard to the second meiotic spindle location; damage of the spindle can cause mistake in the first cleavage divisions. 

However Dumoulin *et al* reported that technical errors has a minor impact on blastocyst development and cannot clearly explain the damaged blastocyst formation after ICSI (3-4, 20). There are considerable increased risk of sex and autosomal chromosome anomalies, and imprinting disorders in ICSI children. So the long term consequences and safety of ICSI are still debate (1, 8, 21-22).

## Conclusion

According to recent study, the routine use of ICSI does not improved fertilization, implantation and chemical pregnancy rates and is not recommended in non-male factor, normozoospermic patients and further large prospective investigations is needed.
