# Corrigendum to “Comparison of conventional IVF versus ICSI in non-male factor, normoresponder patients” [Iran J Reprod Med 2012; 10: 131-136]

**DOI:** 10.18502/ijrm.v19i4.9067

**Published:** 2021-04-22

**Authors:** Maryam Eftekhar, Farnaz Mohammadian, Fariba Yousefnejad, Behnaz Molaei, Abbas Aflatoonian

The authors of the above article published in IJRM, formerly named as “Iranian Journal of Reproductive Medicine (Vol. 10, No. 2; pp: 131–136) have been informed of a misnomer that occurred on page 134 in which the word “randomized” should be replaced with “divided”. On behalf of the author, the publisher wishes to apologize for this error. The online version of article has been updated on April 16, 2021 and can be found at http://journals.ssu.ac.ir/ijrmnew/article-1-265-en.html (doi.org/10.18502/ijrm.vol10i2.9056). 

